# Supplementary material for: Capsid protein structure in Zika virus reveals the flavivirus assembly process
Source: Nat Commun. 2020 Feb 14;11:895. doi: 10.1038/s41467-020-14647-9 (PMC7021721; doi:10.1038/s41467-020-14647-9)
Supplement: Supplementary file 1 — Supplementary Information [file 41467_2020_14647_MOESM1_ESM.pdf]

SUPPLEMENTARY INFORMATION

Capsid protein structure in Zika virus reveals the flavivirus assembly process

Ter Yong Tan*, Guntur Fibriansah*, Victor A. Kostyuchenko, Thiam-Seng Ng, Xin-Xiang Lim, Shuijun Zhang, Xin-Ni Lim, Jiaqi Wang, Jian Shi, Marc C. Morais, Davide Corti, Shee-Mei Lok#

*These authors contribute equally to this work.
#Corresponding author. Email: sheemei.lok@duke-nus.edu.sg

This PDF file includes:

Supplementary Tables 1 to 3

Supplementary Figures 1 to 14

Supplementary References

Supplementary Table 1 | RMSD values of Cα atoms for superposed structures.

		Capsid protomers within dimer			
		1		2	
**PDB ID**		**RMSD (Å)**	**residues***	**RMSD (Å)**	**residues***
1R6R	DENV2 (NMR structure) chain A	3.31	60	3.31	57
	DENV2 (NMR structure) chain B	3.31	60	3.31	57
1SFK	WNV (crystal structure) chain A	3.33	64	2.55	53
	WNV (crystal structure) chain B	3.33	64	2.53	48
5YGH	ZIKV (crystal structure) chain A	2.89	63	2.35	62
	ZIKV (crystal structure) chain B	2.76	62	2.27	61

*number of aligned residues that were used to calculate RMSD

Supplementary Table 2 | Phenix Mtriage summary

**Map resolution estimates**	**E-pr-Fab**	**Capsid**
using map alone (d99)#	8.90	8.08
Overall B_iso	470.00	495.00
comparing with model (d_model)$	8.20	17.00
comparing with model (d_model_b0) b_iso_overall=0	9.60	10.00
d_fsc_model*		
FSC(map,model map)=0	6.97	5.82
FSC(map,model map)=0.143	7.79	7.63
FSC(map,model map)=0.5	9.06	10.65

#d99 - resolution cutoff beyond which Fourier map coefficients are negligeably small. Calculated from the map.
$d_model - resolution cutoff at which the model map is the most similar to the target (experimental) map.
*d_FSC_model - resolution cutoff up to which the model and map Fourier coefficients are similar.

Supplementary Table 3 | Calculated and Experimental molecular weight of ZIKV structural proteins.

**Structural protein**	**Calculated MW (Da)**	**Experimental MW (Da)**
Capsid α1-4	11,648	11,694
Capsid α1-5	13,391	14,947
Envelope	54,267	Not detected
Precursor-membrane	18,695	Not detected

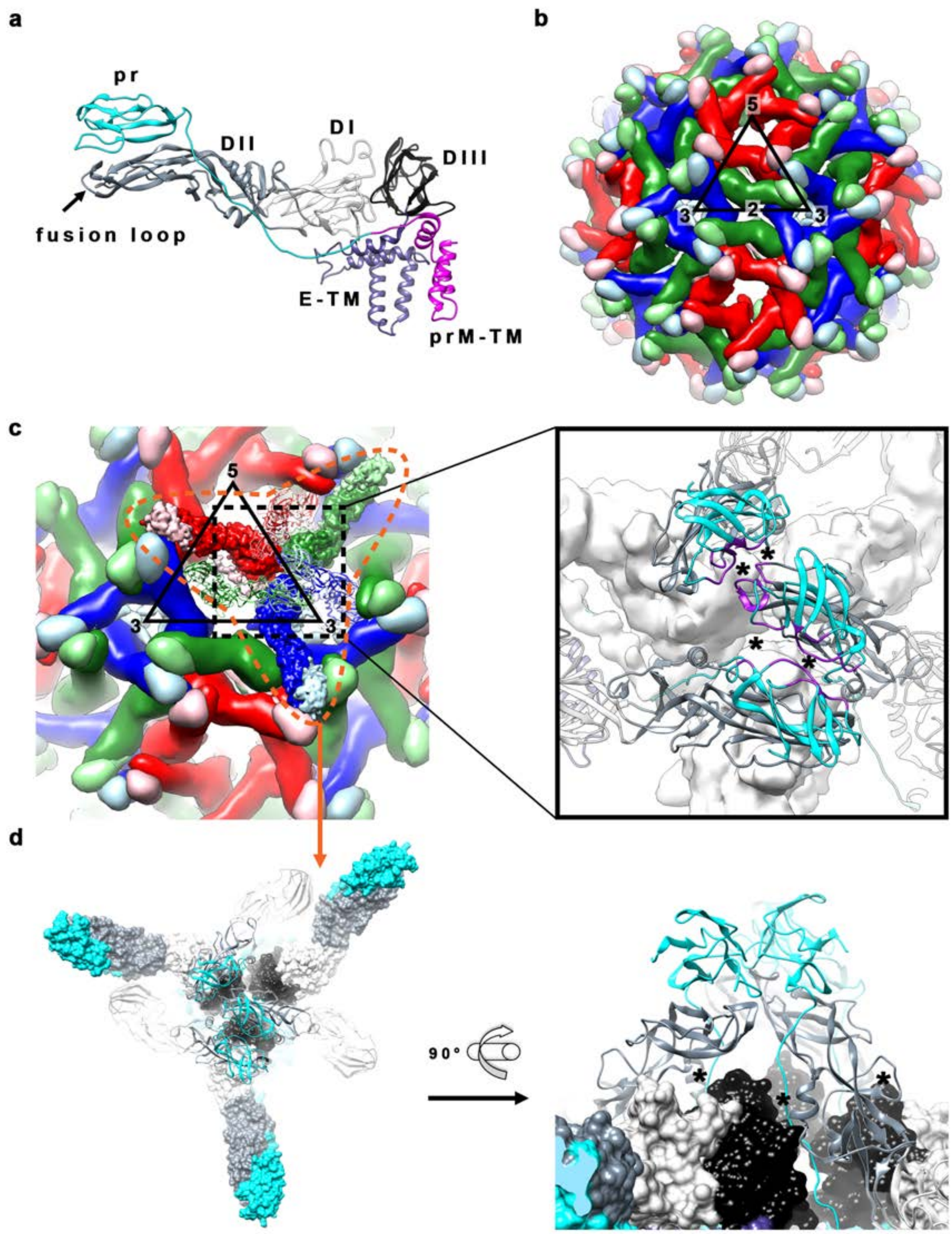

Supplementary Figure 1 | The E and prM proteins organization on immZIKV [1].
a, The structure of an E-prM heterodimer. The E-DI, E-DII and E-DIII are colored in light grey, grey and black, respectively, whereas the pr molecule in cyan. The TM regions of E and prM proteins are colored in purple and magenta, respectively. **b**, The overall structure of an immZIKV particle. An icosahedral asymmetric unit (asu) is indicated by a black triangle. The three individual E proteins in an asu, each located adjacent to either 5-, 2- or 3-fold vertices, are colored in red, green and blue,

respectively and their binding partner, the prM molecule in a lighter shade of the same color. **c**, Zoom-in view of the immZIKV surface. Left panel, each icosahedral asu has a spike (form by E proteins shown as ribbons) located near to 3-fold vertex (dotted black box). Right panel, at the top of the spike, the three E-prM complexes interact between each other (asterisks) [E-DIIs (grey ribbons) and prM molecules (cyan)]. The interacting residues are colored in purple. **d**, Left panel, the top of the trimeric spike (ribbons) also interacts with three E proteins (surfaces) located directly underneath (each of these E proteins originates from a different neighboring spike). Right panel, the interactions are indicated by asterisks.

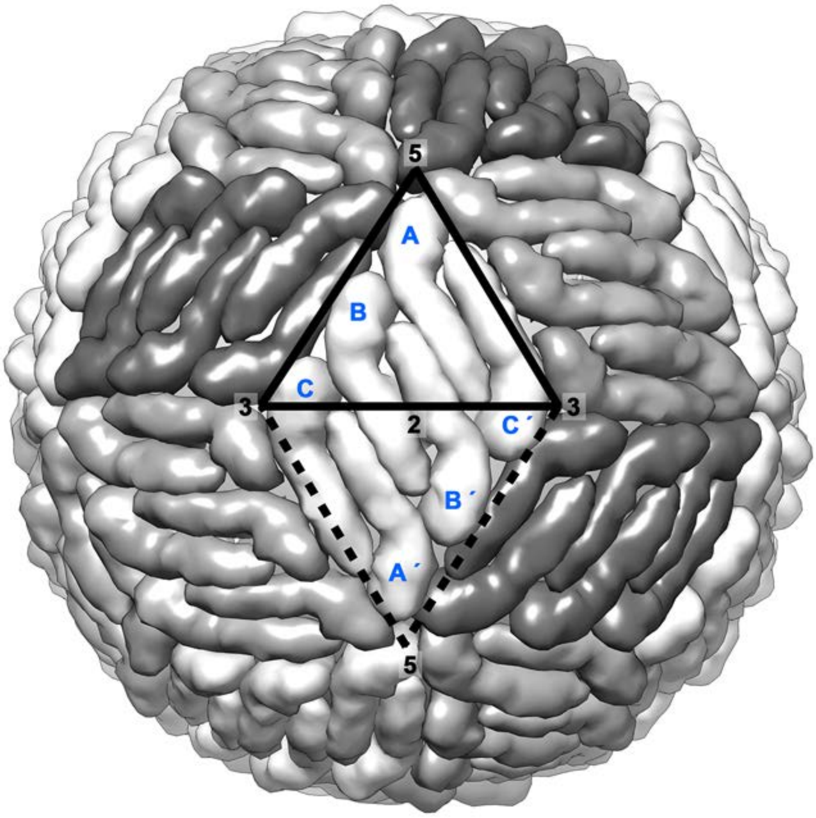

Supplementary Figure 2 | E protein organization on mature ZIKV particle. There are three E protein molecules (molecules A, B and C) in an icosahedral asymmetric unit (asu, black triangle). These E protein molecules and the other three from an adjacent asu (dashed triangle) form a raft structure. In total, there are 30 rafts on the virus particle forming a herringbone structure (shown in a different shade of grey).

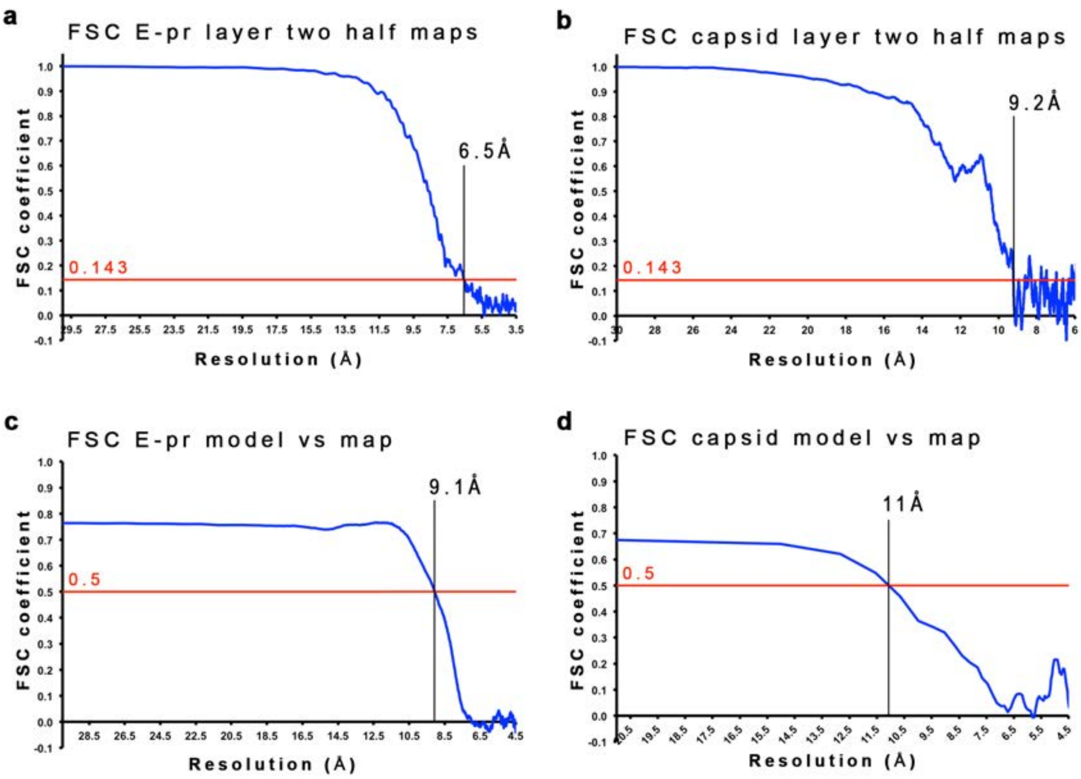

Supplementary Figure 3 | Resolution of the pr-E and capsid layers of immZIKV-Fab DV62.5 map calculated by the gold standard FSC curves, and the FSC between the fitted structure models and their corresponding densities. a-b, The gold standard FSC curve with a 0.143 cut-off between two halves sets of the data showed the resolution of the pr-E layer is 6.5Å (calculated by Phenix mtriage[2]), whereas the capsid layer is 9.2 Å (both calculation using Phenix mtriage[2] and EMAN[3] (Fig. 2a) showed the same resolution). **c-d**, The FSC curves showed the (c) fitted pr-E proteins and (d) capsid models to their corresponding densities at FSC cut-off of 0.5 correlated to ~9.1 and ~10.7 Å, respectively. All FSC curves were calculated using Phenix.mtriage and the statistics of the mtriage results are shown in Supplementary Table 2.

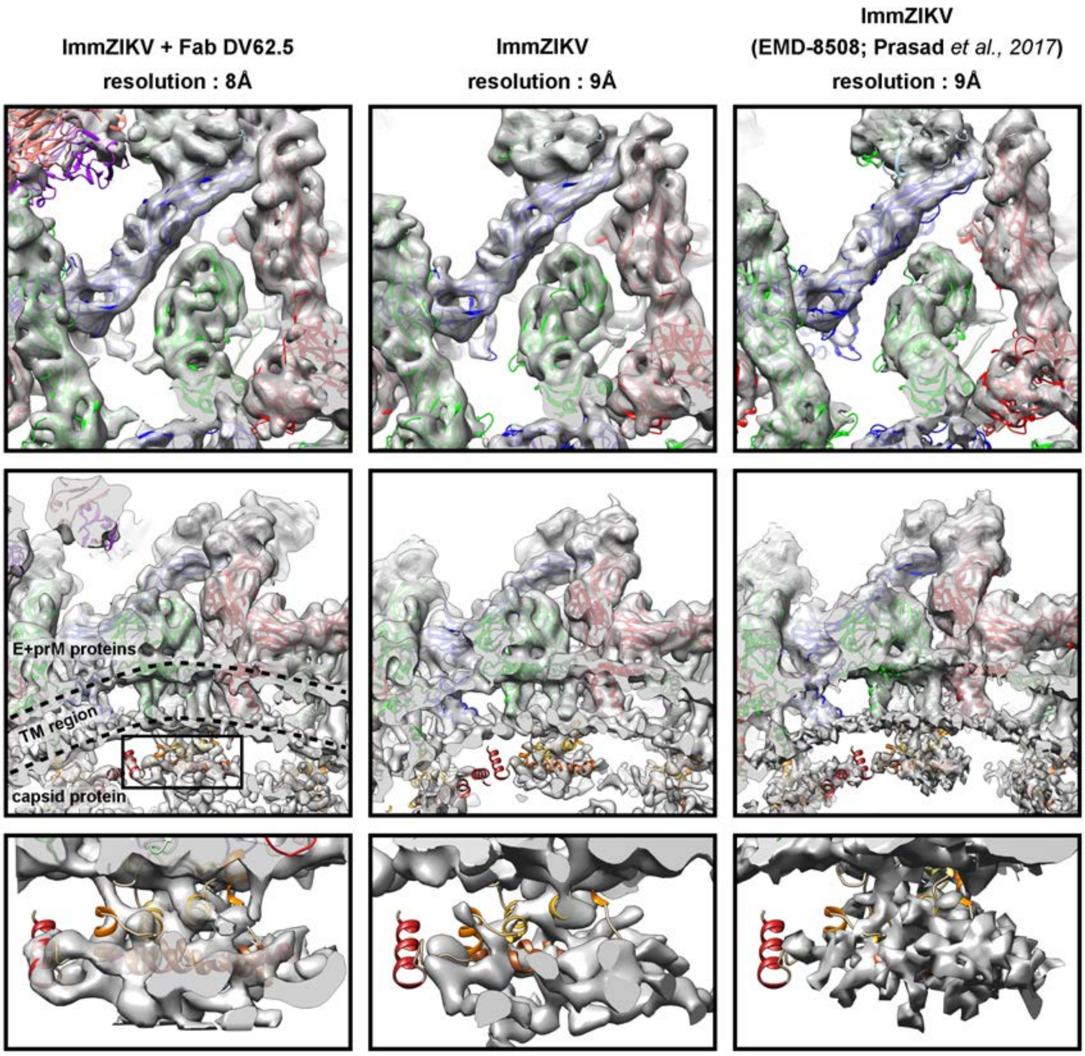

Supplementary Figure 4 | Comparison between the cryoEM density maps of our DV62.5:immZIKV and their uncomplexed structures [EMD-8508 [4]]. Top row, top view of the cryoEM densities corresponding to the prM-E proteins. Middle row, zoom-out view of the cryoEM densities of the E and prM proteins, and the capsid proteins (black box). The bilayer lipid membrane is indicated by dotted lines. The E-prM protein densities, especially around the TM regions, are adjusted to the same thickness. Bottom row, zoom-in view of the capsid dimer densities. For the capsid protein densities, only that in the DV62.5 complexed with immZIKV structure are well resolved.

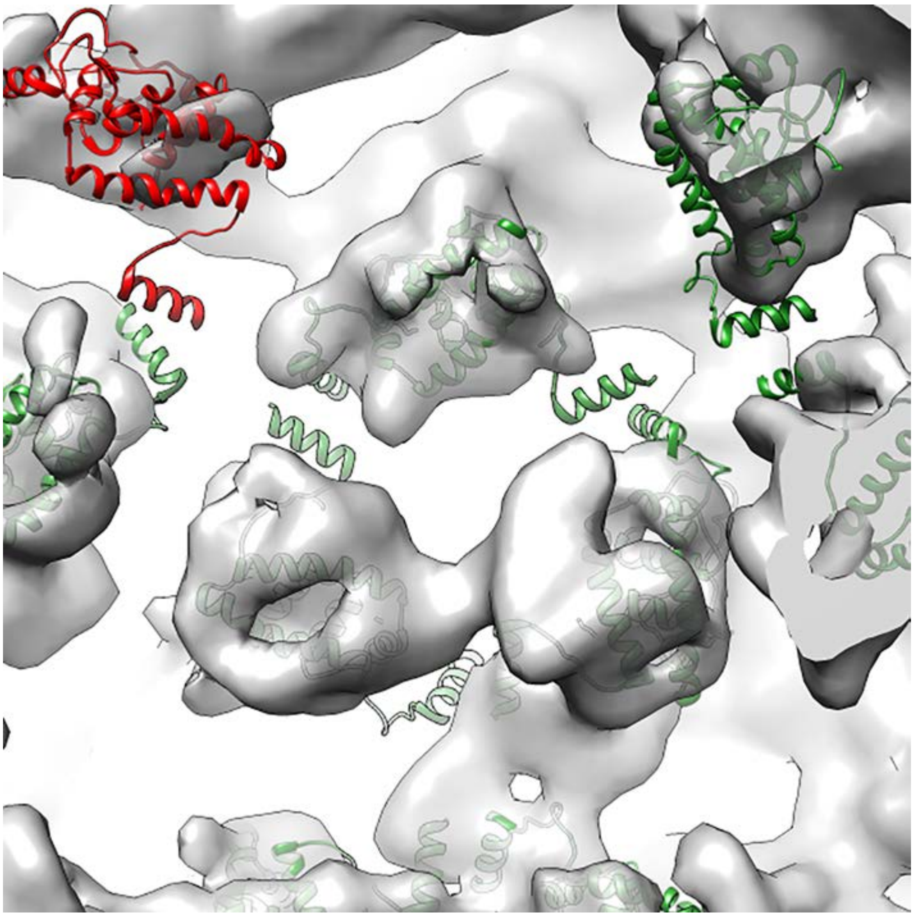

Supplementary Figure 5 | Asymmetric averaged ImmZIKV complexed with DV62.5 tomogram at 16Å resolution showed partial occupancies of capsid protein dimer. Capsid proteins related by icosahedral symmetry are shown as ribbons (both red and green). Capsid proteins that have corresponding densities observed are represented as green ribbons while those without, in red. Density is shown as transparent grey surfaces.

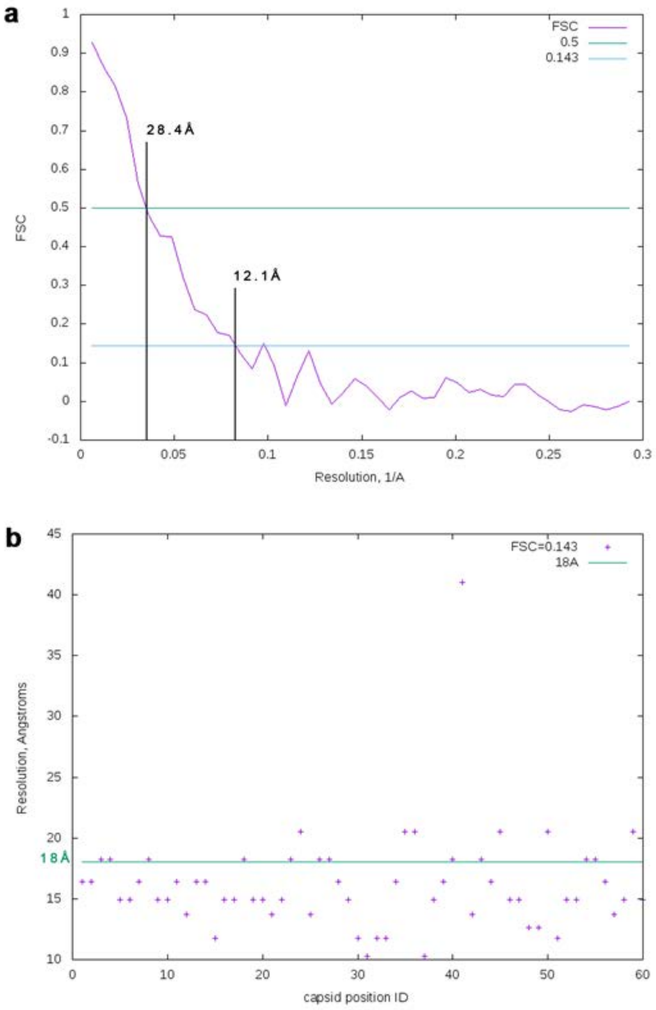

Supplementary Figure 6 | Fourier shell correlation between each of the 60 capsid densities from C1 sub-tomogram averaged and the density from the icosahedral SPA averaged maps. Sixty capsid densities were extracted from the C1 sub-tomogram averaged map and then FSC between each of these capsid densities to that of the icosahedral SPA averaged map was calculated. **a**, An example of one of these FSC curves is shown. **b**, Diagram shows plot of resolutions at the cutoff at 0.143 of the FSC between each of the individual 60 capsid densities to that in the icosahedral averaged SPA map (purple stars). Most are better than 18 Å resolution (green line); the resolution determined by gold-standard FSC of the capsid layer of the C1 sub-tomogram averaged map between two-halves tomograms.

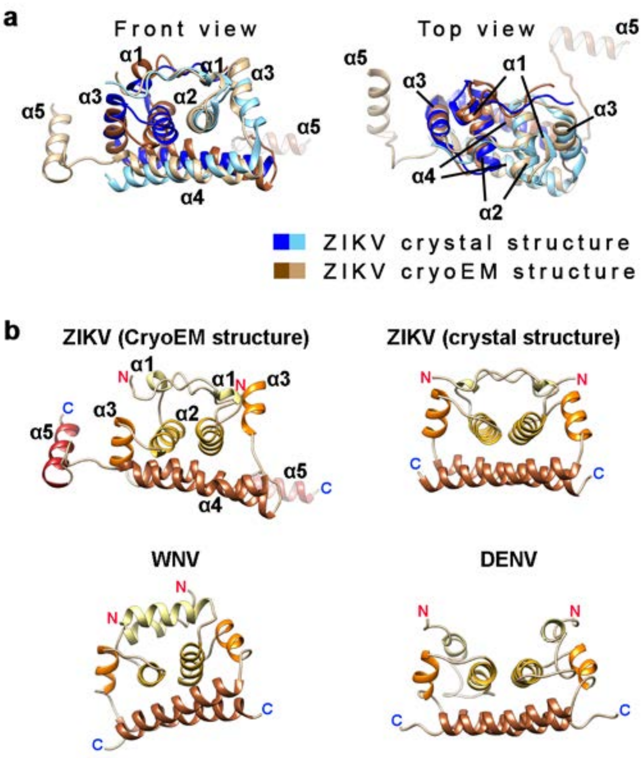

Supplementary Figure 7 | The ZIKV capsid protein tertiary structure. **a**, Superposition of the cryoEM ZIKV capsid protein structure with the crystal ZIKV capsid protein structure. **b**, Structure comparison of the cryoEM ZIKV capsid protein structure with those of the crystal structure of ZIKV [5,6], crystal structure of WNV [7], and NMR structure of DENV [8]. The N-terminus (red N letter) and C-terminus (blue C letter) of the capsid protein are indicated.

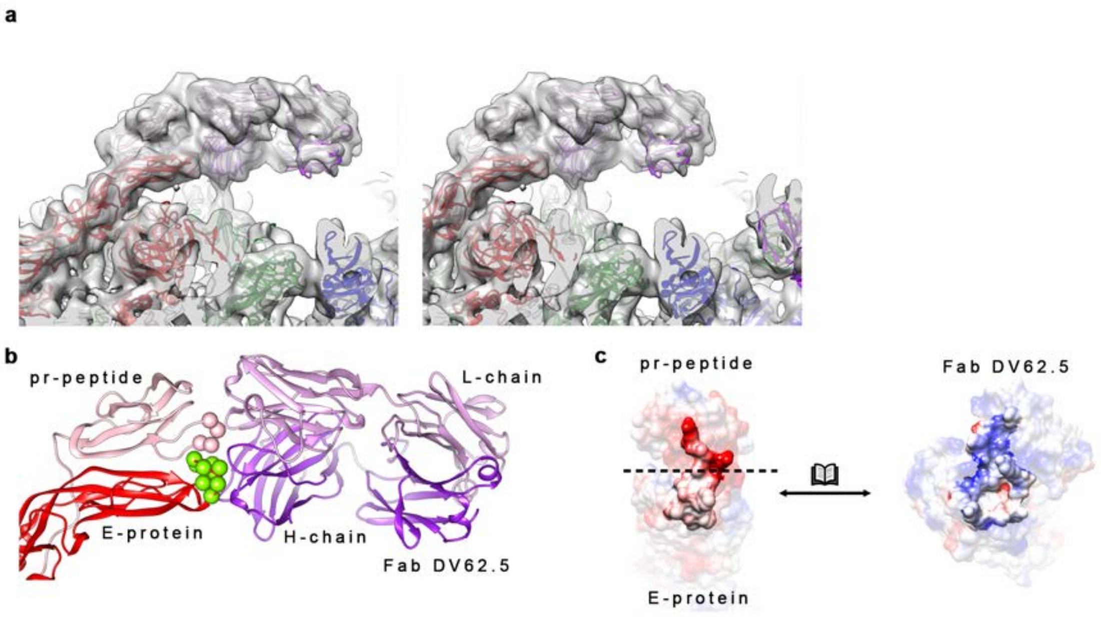

Supplementary Figure 8 | Interactions of Fab DV62.5 with prM-E proteins on the viral surface. a, The fit of Fab DV62.5 and surface proteins into the immZIKV:DV62.5 map. The fitted Fab DV62.5 (light purple) binding to red E protein molecule is shown in stereo view. **b**, The DV62.5 epitope (spheres) is located across the fusion loop (green spheres) of an E protein and its adjacent pr-peptide. **c**, Open-book representation showing the surface charges of the epitope and Fab paratope are complementary. The positive, negative charges and the hydrophobic surfaces are colored in blue, red and white, respectively.

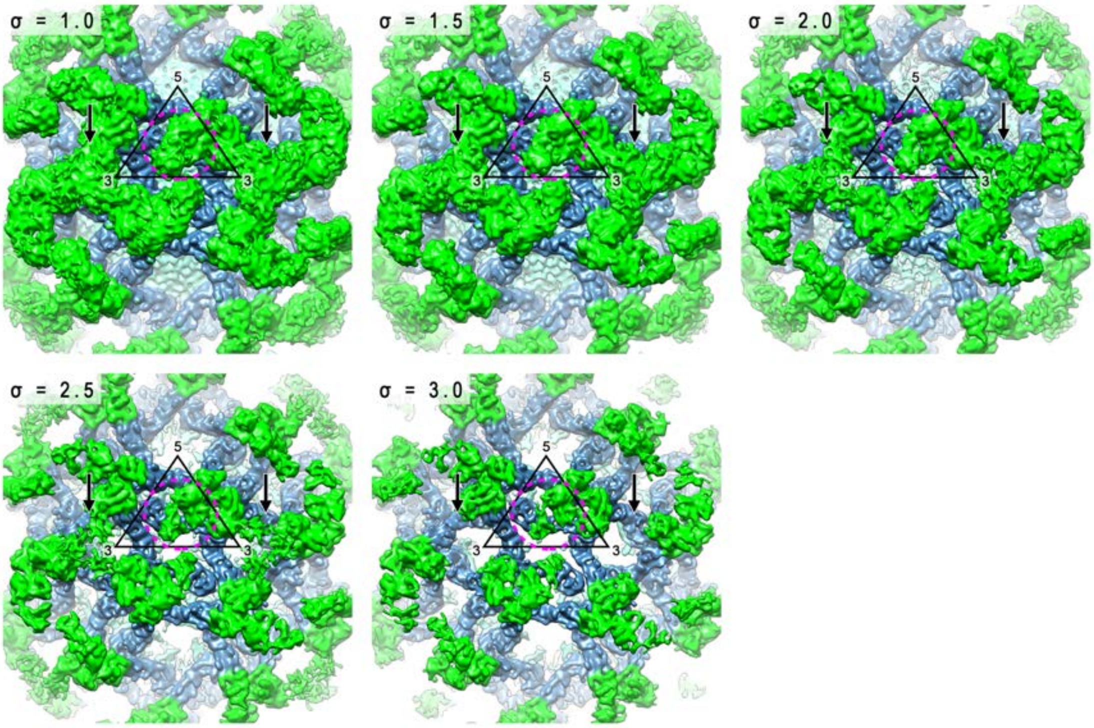

Supplementary Figure 9 | Fab DV62.5 occupancies on the surface of immZIKV. **T**he variable region of the Fab DV62.5 densities (magenta circle) binding to red E protein (Fig. 2d) are as strong as the E protein densities suggesting full occupancy. On the other hand, the Fab DV62.5 densities around the 3-fold vertices (indicated by black arrow) are weaker indicating partial occupancies. CryoEM density map displayed at different contour levels are shown.

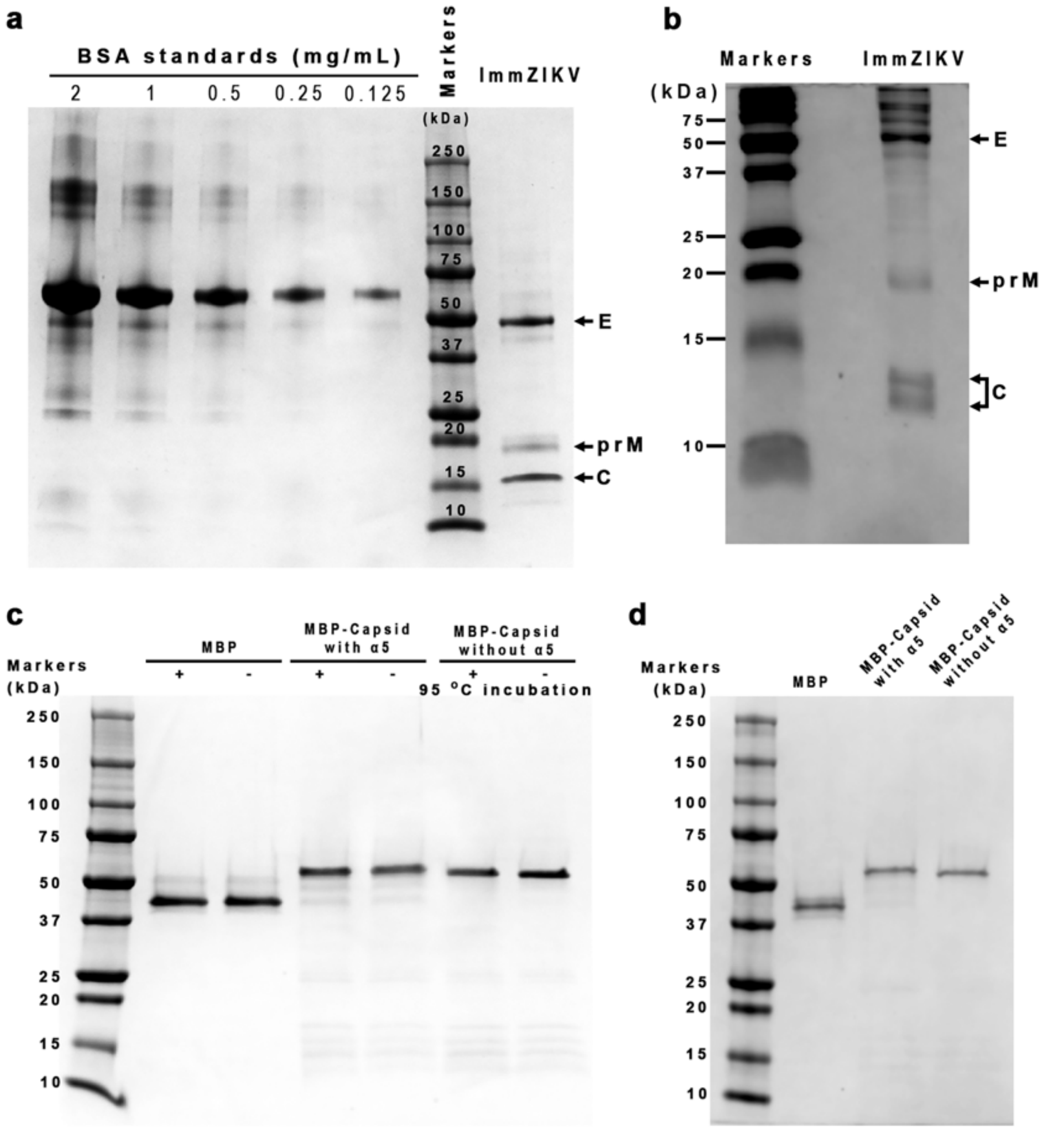

Supplementary Figure 10 | Coomassie blue stained SDS–PAGE gel profile of purified immZIKV and the purified recombinant MBP-capsid fusion proteins. a, The immZIKV preparation showed clear bands of E, prM and capsid proteins in a 4 – 20% gradient gel. There is no band that corresponds to M protein (8,415 Da) indicating the virus preparation was highly immature. **b**, In a higher percentage gel (15%), two capsid bands at around 13 kDa were observed. **c**, SDS-PAGE of the recombinant MBP-capsid fusion proteins. Samples were either boiled (+) or not-boiled(-). MBP only, MBP:capsid with helix α5 and MBP:capsid without helix α5 were separated on a 4-20% gradient gel. By Coomassie blue, only one band showing monomeric molecular weight of all samples was detected. Comparison of the intensities of the bands of all samples showed similar amounts had been loaded as in Fig. 4b. **d**, SDS-PAGE of the recombinant MBP-capsid fusion proteins. This is to

show that similar amount of proteins had been loaded in the native-PAGE as in Fig. 4c.

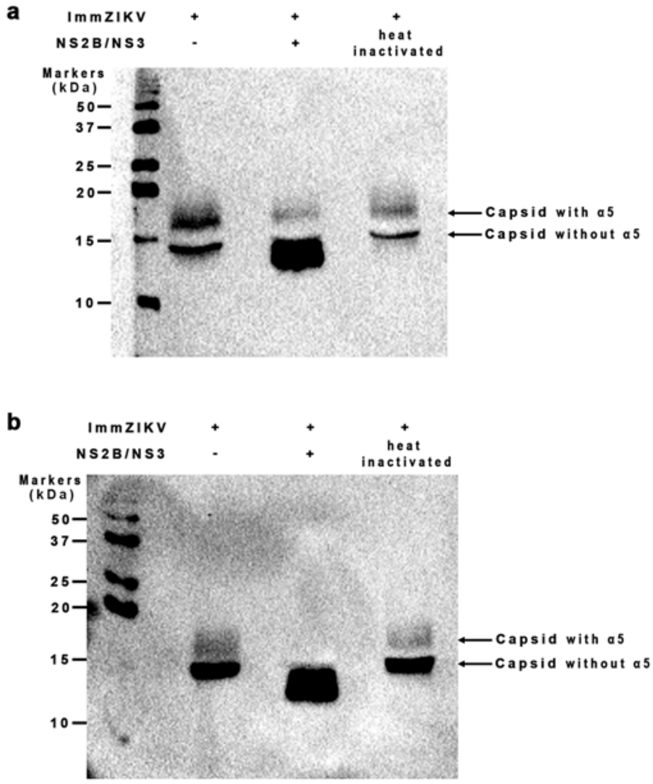

Supplementary Figure 11 | Two experimental replicates for the cleavage of helix α5 from the capsid protein by NS2B/NS3. The two replicates were done on capsid protein from two different virus preparations using the same protocol used in the experiment shown in Fig. 4a.

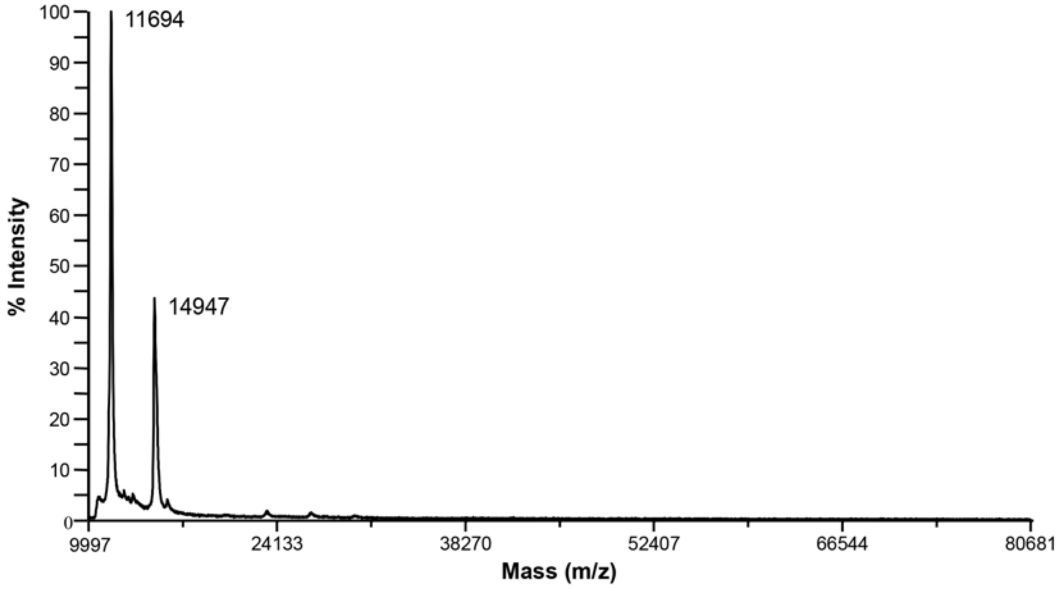

Supplementary Figure 12 | Mass spectrometry detects presence of capsid protein with helix α5. MALDI-TOF mass spectrum of intact immZIKV using α-Cyano-4-hydroxycinnamic acid as the matrix. The virus sample was treated with acetonitrile and trifluoroacetic acid prior to centrifugation and only the supernatant was spotted on the MALDI plate. Using this method, only two peaks that correspond to capsid protein were observed. The membrane anchored proteins (E and prM proteins) aggregated, thus no peaks corresponding to E and prM proteins were observed. This is similar to that observed in the mass spectrometry studies on intact Sindbis virus [9]. The peak at 11,694 Da corresponds to capsid protein that contains residues ~ 1-103 (containing N-terminus to end of helix α4), whereas peak at 14,947 Da corresponds to capsid protein that contains residues ~ 1-123 (N-terminus to the end of helix α5) likely with two molecules of phospholipid (~700 Da each).

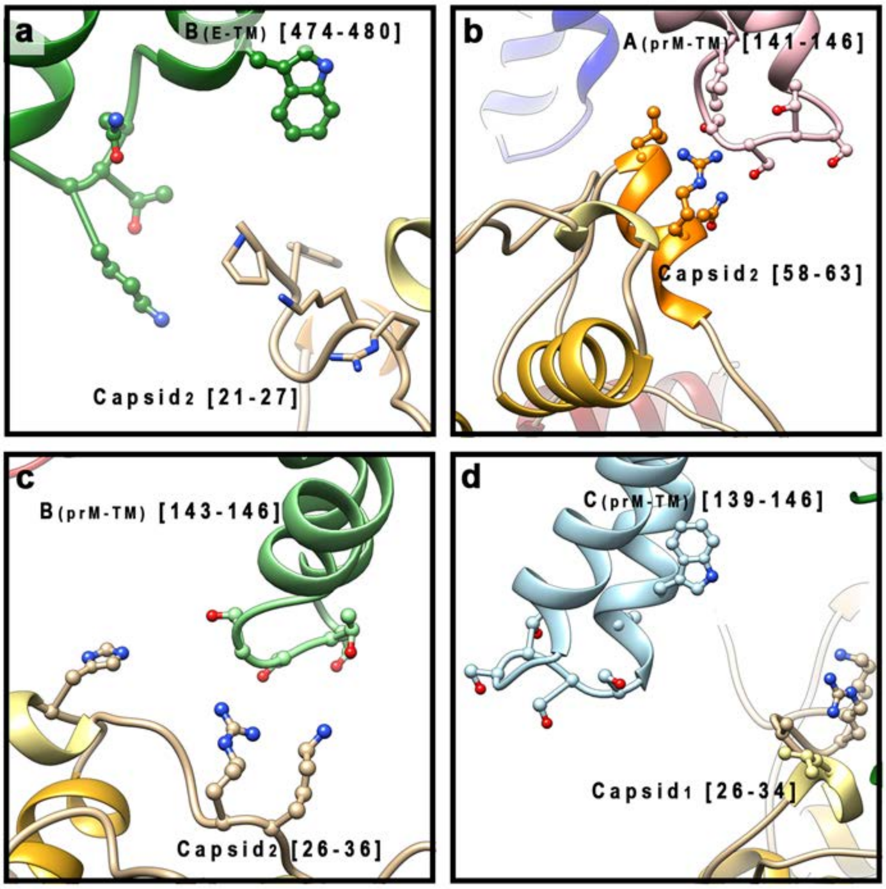

Supplementary Figure 13 | Possible interactions of the capsid dimer with the other components in the virus particle. a-d, The capsid dimer likely interacts with the green E protein TM and three prM TM (light pink, light green and light blue) regions. Examination of residues in these TM regions of E and prM proteins showed charge complementary with that on the capsid protein.

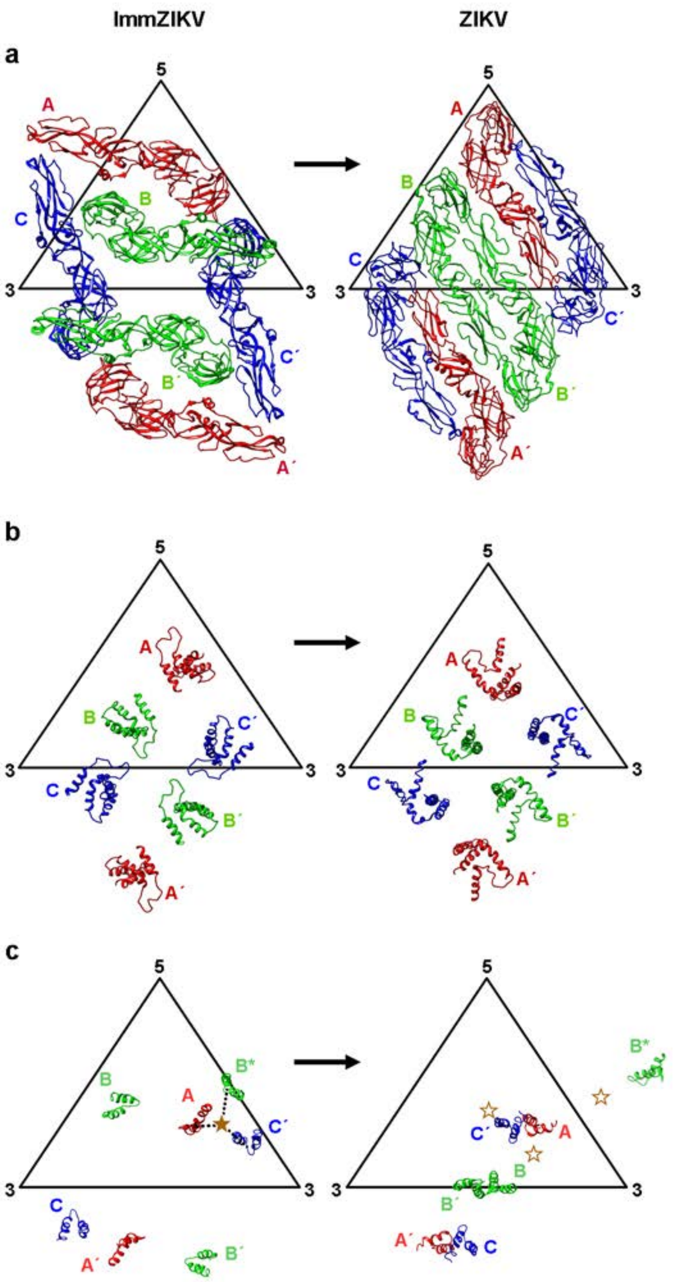

Supplementary Figure 14 | The maturation process of immZIKV results in the loss of icosahedral symmetry organization of the capsid dimer proteins. a-b, By comparing the immature (left panels) and mature (right panels) flaviviruses, it has been determined previously that the maturation process causes dramatic reorganization of the E protein ectodomains (**a**), while E-TM regions (**b**) stayed in approximately the same position [1]. **c**, In contrast, the TM positions of the prM protein in the immature and the M protein in mature flavivirus changes dramatically [1,10]. Our capsid protein cryoEM structure (filled brown star) in immZIKV shows it interacts mostly with the TM regions of three prM proteins (dotted black line). During the maturation process, the capsid protein may move together with one of these three prM

molecules (empty brown stars) in an asymmetric unit in the mature virus. The different movements of capsid protein dimers in each of the 60 asymmetric units within a particle will lead to a loss of icosahedral symmetry.

Supplementary References

1. Kostyuchenko, V.A., Zhang, Q., Tan, J.L., Ng, T.S. & Lok, S.M. Immature and mature dengue serotype 1 virus structures provide insight into the maturation process. *J Virol* **87**, 7700-7 (2013).

2. Afonine, P.V. et al. New tools for the analysis and validation of cryo-EM maps and atomic models. *Acta Crystallogr D Struct Biol* **74**, 814-840 (2018).

3. Ludtke, S.J., Baldwin, P.R. & Chiu, W. EMAN: semiautomated software for high-resolution single-particle reconstructions. *J Struct Biol* **128**, 82-97 (1999).

4. Prasad, V.M. et al. Structure of the immature Zika virus at 9 A resolution. *Nat Struct Mol Biol* **24**, 184-186 (2017).

5. Li, T. et al. Structural insight into the Zika virus capsid encapsulating the viral genome. *Cell Res* **28**, 497-499 (2018).

6. Shang, Z., Song, H., Shi, Y., Qi, J. & Gao, G.F. Crystal Structure of the Capsid Protein from Zika Virus. *J Mol Biol* **430**, 948-962 (2018).

7. Dokland, T. et al. West Nile virus core protein; tetramer structure and ribbon formation. *Structure* **12**, 1157-63 (2004).

8. Ma, L., Jones, C.T., Groesch, T.D., Kuhn, R.J. & Post, C.B. Solution structure of dengue virus capsid protein reveals another fold. *Proc Natl Acad Sci U S A* **101**, 3414-9 (2004).

9. Kim, Y.J., Freas, A. & Fenselau, C. Analysis of viral glycoproteins by MALDI-TOF mass spectrometry. *Anal Chem* **73**, 1544-8 (2001).

10. Kostyuchenko, V.A. et al. Structure of the thermally stable Zika virus. *Nature* **533**, 425-8 (2016).
