# Supplementary material for: Capsid protein structure in Zika virus reveals the flavivirus assembly process
Source: Nat Commun. 2020 Feb 14;11:895. doi: 10.1038/s41467-020-14647-9 (PMC7021721; doi:10.1038/s41467-020-14647-9)
Supplement: Supplementary file 3 — Reporting Summary [file 41467_2020_14647_MOESM3_ESM.pdf]

nature research

Corresponding author(s): Shee-Mei Lok

Last updated by author(s): Dec 26, 2019

Reporting Summary

Nature Research wishes to improve the reproducibility of the work that we publish. This form provides structure for consistency and transparency in reporting. For further information on Nature Research policies, see Authors & Referees and the Editorial Policy Checklist .

Statistics

For all statistical analyses, confirm that the following items are present in the figure legend, table legend, main text, or Methods section.

n/a	Confirmed	
☒	☐	The exact sample size (n) for each experimental group/condition, given as a discrete number and unit of measurement
☒	☐	A statement on whether measurements were taken from distinct samples or whether the same sample was measured repeatedly
☒	☐	The statistical test(s) used AND whether they are one- or two-sided<br>*Only common tests should be described solely by name; describe more complex techniques in the Methods section.*
☒	☐	A description of all covariates tested
☒	☐	A description of any assumptions or corrections, such as tests of normality and adjustment for multiple comparisons
☒	☐	A full description of the statistical parameters including central tendency (e.g. means) or other basic estimates (e.g. regression coefficient) AND variation (e.g. standard deviation) or associated estimates of uncertainty (e.g. confidence intervals)
☒	☐	For null hypothesis testing, the test statistic (e.g. F, t, r) with confidence intervals, effect sizes, degrees of freedom and P value noted<br>*Give P values as exact values whenever suitable.*
☒	☐	For Bayesian analysis, information on the choice of priors and Markov chain Monte Carlo settings
☒	☐	For hierarchical and complex designs, identification of the appropriate level for tests and full reporting of outcomes
☒	☐	Estimates of effect sizes (e.g. Cohen's d, Pearson's r), indicating how they were calculated

Our web collection on statistics for biologists contains articles on many of the points above.

Software and code

Policy information about availability of computer code

Data collection	Leginon
Data analysis	Gctf, EMAN2.1, Relion 2.1, Chimera, COOT, Phenix 1.16, IMOD, emClarity

For manuscripts utilizing custom algorithms or software that are central to the research but not yet described in published literature, software must be made available to editors/reviewers. We strongly encourage code deposition in a community repository (e.g. GitHub). See the Nature Research guidelines for submitting code & software for further information.

Data

Policy information about availability of data

All manuscripts must include a data availability statement. This statement should provide the following information, where applicable:

- Accession codes, unique identifiers, or web links for publicly available datasets
- A list of figures that have associated raw data
- A description of any restrictions on data availability

The cryo-EM maps of immZIKV-Fab DV62.5 complex and uncomplexed immZIKV have been deposited in the Electron Microscopy Database (EMDB). The coordinates of immZIKV-Fab DV62.5 complex and uncomplexed immZIKV have been deposited in the Protein Data Bank (PDB). The cryoEM subtomo averaged map of immZIKV-Fab DV62.5 has also been deposited in the EMDB. The respective accession codes of the data have been indicated in the final accepted manuscript (in the Data Availability section).

Field-specific reporting

Please select the one below that is the best fit for your research. If you are not sure, read the appropriate sections before making your selection.

Life sciences study design

All studies must disclose on these points even when the disclosure is negative.

Sample size	N/A
Data exclusions	N/A
Replication	N/A
Randomization	N/A
Blinding	N/A

Reporting for specific materials, systems and methods

We require information from authors about some types of materials, experimental systems and methods used in many studies. Here, indicate whether each material, system or method listed is relevant to your study. If you are not sure if a list item applies to your research, read the appropriate section before selecting a response.

Materials & experimental systems

n/a	Involved in the study	
☐	☒	Antibodies
☐	☒	Eukaryotic cell lines
☒	☐	Palaeontology
☒	☐	Animals and other organisms
☒	☐	Human research participants
☒	☐	Clinical data

Methods

n/a	Involved in the study	
☒	☐	ChIP-seq
☒	☐	Flow cytometry
☒	☐	MRI-based neuroimaging

Antibodies

Antibodies used

1. DV62.5 was isolated and purified by Davide Corti's laboratory. For more information refer to Beltramello, M. et al. 2010.
2. Anti-MBP antibody: NEB, Catalog no #E8032S, Lot no 0101705
3. Anti-Zika virus capsid protein antibody: GeneTex, Catalog no GTX133317, Lot no 42921
4. Gt anti-mouse IgG (H+L) secondary antibody, HRP conjugate: Invitrogen, Catalog no # A16072
5. Gt anti-rabbit IgG (H+L) secondary antibody, HRP conjugate: Invitrogen, Catalog no # 65-6120, Lot no TE268257

Validation

1. DV62.5 is a human monoclonal antibody first isolated and characterized by Beltramello, M. et al. 2010.
2. Anti-MBP antibody from NEB is a murine anti-maltose binding protein (MBP) antibody, isotype IgG2a. Verified by manufacturer for use in both Western blotting and ELISA. (https://www.neb.sg/products/e8032-anti-mbp-monoclonal-antibody#Product%20Information)
3. Anti-Zika virus capsid protein antibody from GeneTex has been verified by manufacturer for use in Western blotting and immunofluorescence. (https://www.genetex.com/Product/Detail/Zika-virus-Capsid-protein-antibody/GTX133317)

Eukaryotic cell lines

Policy information about cell lines

Cell line source(s)	C6/36 from ATCC
Authentication	C6/36 was purchased and authenticated by ATCC.
Mycoplasma contamination	We followed the protocol published by Young et al., 2010 and did not detect any mycoplasma contamination in our cell cultures.
Commonly misidentified lines (See ICLAC register)	None listed on latest version 9 of the register of misidentified cell lines
