# Supplementary material for: Capsid protein structure in Zika virus reveals the flavivirus assembly process
Source: Nat Commun. 2020 Feb 14;11:895. doi: 10.1038/s41467-020-14647-9 (PMC7021721; doi:10.1038/s41467-020-14647-9)
Supplement: Supplementary file 4 — Description of Additional Supplementary Files [file 41467_2020_14647_MOESM4_ESM.pdf]

**Description of Additional Supplementary Files**

File Name: Supplementary Movie 1
Description: **The fit of the capsid dimer into its corresponding density in the cryoEM immZIKV:DV62.5 complex map.**

File Name: Supplementary Movie 2
Description: **Asymmetric averaged tomogram showing the occupancies of capsid dimers at the RNAviral lipid membrane interface is not full.** Capsid proteins related by icosahedral symmetry are shown as ribbons (both red and green). Capsid proteins that have corresponding densities observed are represented as green ribbons while those without, in red. Density is shown as transparent grey surfaces.

File Name: Supplementary Movie 3
Description: **The capsid protein dimers assemble basic building blocks for the architecture of the flavivirus particle.**
